# The effect of immunonutrition on tumor infiltrative t lymphocytes and regulatory t cells in rectal tumor patients receiving neoadjuvant chemoradiotherapy: a prospective randomized clinical study

**DOI:** 10.55730/1300-0144.5408

**Published:** 2022-06-18

**Authors:** Mehmet Onur GÜL, Cebrail AKYÜZ, Selvinaz ÖZKARA

**Affiliations:** 1 Department of Surgical Oncology, Çukurova University, Balcalı Training and Research Hospital, Adana, Turkey; 2 Department of Gastroenterologic Surgery, Haydarpaşa Numune Training and Research Hospital, İstanbul, Turkey; 3 Department of Pathology, Haydarpaşa Numune Training and Research Hospital, İstanbul, Turkey

**Keywords:** Colorectal cancer, nutrition, regulatory t cells, tumor infiltrative lymphocytes

## Abstract

**Background/aim:**

The rationale behind using immunonutrition in cancer patients is to prevent malnutrition, manage the host’s immune response, and keep cancer under control by utilizing the potential immune system available in the host against the tumor. This prospective- study aims to assess the impact of immunonutrition on tumor-infiltrating lymphocytes (TILs) and regulatory T cells (Tregs) in rectal cancer patients receiving neoadjuvant chemoradiotherapy.

**Materials and methods:**

This is a single-center, prospective study. Forty patients diagnosed with middle and lower rectal tumors were enrolled in the study between March 2018 and December 2019. Nutrition protocols were given to all study subjects prior to surgery. Tissue CD4, CD8, and Fox P3 expression prior to enrollment (endoscopic biopsy specimens) and following surgery (resected tissue) were compared.

**Results:**

Longitudinal data was available for 30 patients. In the present study, 15 patients were given immuno-nutrition, and 15 patients received standard nutrition. The immunonutrition and standard nutrition groups were similar regarding CD4 [10 (5–20) vs. 10 (10–10), p = 0.653], CD8 [30 (20–35) vs. 30 (20–40), p = 0.870], lymphocyte counts [2 (2–3) vs. 2 (2–3), p = 0.325], fox p3 value [10 (10–10) vs. 10 (10–10), p = 0.775], and CD4/CD8 ratio [0.33 (0.29–0.66) vs. 0.50 (0.29–0.50), p = 0.870] on endoscopic biopsy. CD4 [10 (7.5–25) vs. 30 (10–50), p = 0.050], CD8 [60 (40–60) vs. 50 (40–60), p = 0.713] and Fox P3 [10 (5–10) vs. 10 (2.5–10), p = 0.935] were also similar in tissues extracted by surgery. However, the standard nutrition group had significantly higher CD4/CD8 values in their tissues removed on surgery [0.25 (0.14–0.50) vs. 0.66 (0.28–1), p = 0.026]

**Conclusion:**

The present study revealed that CD4/CD8 ratios were lower in the immunonutrition group in comparison to the group receiving standard nutritional supplements before surgery.

## 1. Introduction

Neoadjuvant chemoradiotherapy (CRT) increases the chance of resection by decreasing the tumor volume in locally advanced rectal cancers and reducing local recurrence [1,2]. After neoadjuvant chemoradiotherapy, a complete pathological response is obtained in 20% of the patients in histopathological evaluations. It is linked with a reduced possibility of distant recurrences and improved long-term survival [3]. In addition to neoadjuvant CRT, another factor affecting the clinical outlook of patients with rectal cancer is malnutrition. The importance of undernutrition has been widely reported for many years and varies between 19% and 80%, depending on the country and patient subset examined [4,5]. The prevalence of malnutrition in patients with colorectal cancer (CRC) has been reported to vary between 45% and 60% [6].

Neoadjuvant CRT and nutrition affect the immune system, and they can eradicate tumor cells or curb tumor growth [7]. It is known that tumor cells and the immune system have a reciprocal relationship, which plays an important part in tumor growth [8–10].

It has been suggested that cytotoxic agents and radiation may provide antitumor activities by inducing the antitumor immune system [11]. However, chemoradiotherapy is rarely sufficient to induce a therapeutically significant antitumor immune response, as it can also induce activation of immune suppressive pathways [12]. The mechanism is not very clear yet. Significant components of the immune system are myeloid cells, lymphocytes, cytokines, and chemokines. Among them, tumor-infiltrating lymphocytes (TILs) and regulatory T cells (Tregs) indicate tumor immunogenicity, and their combination is linked with how the immune system reacts [10]. In general, TILs have been reported to be associated with suppressing antitumor immunity by CD8 + cytotoxic T lymphocytes (CTLs) and CD4 + Th1 cells. In contrast, FOXP3 + regulatory T cells (Tregs) are involved in effective antitumor immunity [13]. However, a few studies have shown a connection between the existence of Foxp3 + cells in the surrounding area of the tumor in colorectal cancer and a favorable survival [11,12].

The primary rationale behind using immunonutrition in cancer cases is to manage cancer by utilizing the body’s defense system against the tumor to regulate the host’s immune response and prevent malnutrition [14]. However, the observed effects are challenging to interpret. Therefore, the objective of this study was to determine the effect of immunonutrition on TILs and Tregs in rectal cancer patients receiving neoadjuvant chemoradiotherapy. Tissue CD4, CD8, and Fox P3 expression prior to enrollment (endoscopic biopsy specimens) and following surgery (resected tissue) were compared with immunohistochemistry.

## 2. Materials and methods

### 2.1. Study design

**Figure 1:**
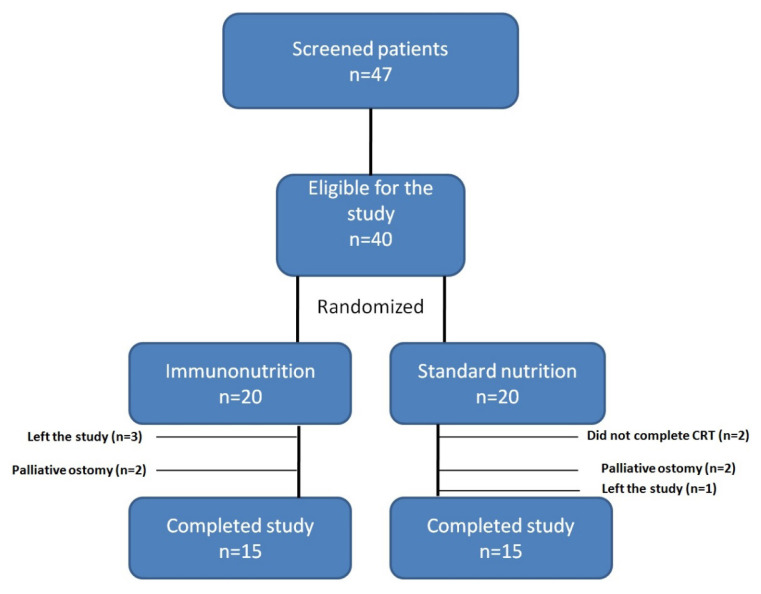
CONSORT diagram demonstrating patient enrollment.

The prospective, single-center study was conducted between March 2018 and December 2019. Approval was obtained from the ethics committee (HNEAH-KAEK2018/KK/07) for this study. Patients younger than 18 years old (n: 1), on dialysis due to kidney failure (n: 2), with uncontrolled infection (n: 1), with diabetes (n: 3), with severe psychiatric disease (n: 0), undergoing palliative procedures and not complying with nutritional therapy were not included into the study population. All patients were initially evaluated with anthropometric methods, including deviation from ideal body weight and NRS 2002 score to exclude severe malnutrition. The study population comprised 40 patients diagnosed with middle and lower rectal tumors and recommended CRT according to NCCN clinical practice guidelines at the time of the study [15]. Written informed consent was obtained from all participants. A computer-generated randomization was applied by an independent research assistant in a 1:1 allocation in the blocks of varying sizes after the participants were prestratified for exclusion criteria. Forty patients were assigned into one of the two groups, [immunonutrition group (n = 20) and standard nutritional supplement group (n = 20)]. Two patients could not complete the long-term neoadjuvant CRT during the study period, four patients underwent palliative ostomy, and four patients were excluded from the study due to incompatibility. CONSORT diagrams demonstrating patient enrollment are given in Figure 1.

### 2.2. General treatment

Neoadjuvant radiotherapy involved 50.4 Gy given in 28 fractions for five weeks. Simultaneously, fluoropyrimidine-based chemotherapy including 5-FU or oral capecitabine was administered through the intravenous route. Surgical resection of the primary tumor was carried out 6–8 weeks after CRT was completed. The patients were applied to the hospital 7th day before the preoperative period, their oral intake was arranged, the use of formulas and their diets were explained, and they were followed up. The patients whose preoperative examinations continued and required hospitalization were hospitalized and their food intake and oral intake were followed up in the hospital. The remaining patients were followed up by phone calls daily, and they were hospitalized 2 days prior to surgery. To assess the nutritional status of the patients, body mass index (BMI) and nutritional risk score (NRS 2002), serum prealbumin and albumin levels were measured (ELISA) 7 days and 1 day prior to surgery and at 2^nd^ and 7^th^ postoperative days [16]. While the control group was given standard diet including about 1900 kilocalories of energy per day, 85 g of protein, 80 mg of vitamin C, 1000 mg of calcium, 10 mg of iron and 10 mg of zinc and standard oral nutritional supplements, the immunonutrition group was given immunomodulating substrates in addition to the standard diet. Unlike standard nutritional solutions, immune nutrition solutions also contained arginine, omega 3 fatty acids and nucleotides. The Immunonutrition group was given an immunonutrition supplement thrice daily. In standard oral nutritional supplements, foods that do not contain immunonutrition were used three times a day. Standard enteral formulas have a composition that meets the nutritional and caloric needs of the general population [17]. They may have a standard nutrient profile or be nutrients adapted for certain conditions or diseases. All patients underwent surgery by the same surgical team experienced in onco-surgery.

All subjects underwent endoscopic examination and tissue sampling prior to CRT. All endoscopic procedures and tissue sampling were carried out by (C.A, E.G). Biopsy specimens collected on the endoscopic examination before neoadjuvant CRT and resection materials in the postoperative period were compared histopathologically for TILs and FOXp3 levels.

### 2.3. Histopathologic examination

**Figure 2:**
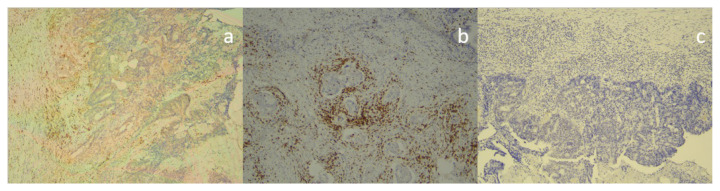
Immunohistochemical staining for CD4, CD8, and Foxp3 in rectal cancer tissues (×100): (a) CD4 + cells stained with monoclonal antibody. (b) CD 8+ cells stained with monoclonal antibody. (c) Foxp3 + cells stained with monoclonal antibody.

**Figure 3:**
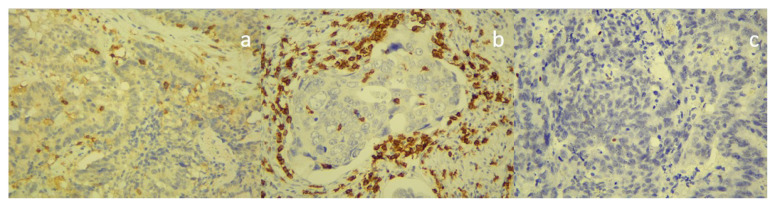
Immunohistochemical staining for CD4, CD8, and Foxp3 in rectal cancer tissues (×40): (a) CD4 + cells stained with monoclonal antibody. (b) CD 8+ cells stained with monoclonal antibody. (c) Foxp3 + cells stained with monoclonal antibody.

Endoscopic biopsy materials obtained from colonoscopy samples were fixed with buffered formaldehyde solution. It was passed through routine tissue traces and embedded in paraffin blocks. Preparations obtained from 2–4 micron sections and embedded into paraffin blocks were stained with hematoxylin-eosin (H&E) and exposed to immunohistochemical staining in a fully automated stainer with the monoclonal antibody (Figures 2a, 2b, 2c, 3a, 3b, 3c) (CD4: Mouse monoclonal clone: 4B12 1/100 dilution, Preprogrammed Leica^®^ BOND™, Leica Biosystems, Wetzlar, Germany, CD8: Mouse monoclonal clone: 4b11 1/100 dilution, Preprogrammed Leica^®^ BOND™, Leica Biosystems, Wetzlar, Germany,, FOX p3: EpitoRabbit monoclonal clone: ep340 1/150 dilution, Epitomics Inc., Burlingame, CA, USA). Later, H&E and immunohistochemically stained preparations were examined under light microscopy under 40X and 100X magnification. During the evaluation, the granulation tissue areas belonging to the ulcer surface and the ulcer base were excluded. Five different areas with the densest lymphocytes under 400 × magnification were counted in tumor infiltration foci. Only intraepithelial lymphocytes in the tumor region were considered tumor-infiltrating lymphocytes. The “hot spot” method, which consists of selecting the areas with the most abundant lymphocytic infiltration on the entire section surface, was used in the evaluation. The number of TILs expressing each antigen was determined as the mean value of counts in each of five representative high-power microscopic felds (N.A: 0.65 mm, W.D: 0.6 mm, Olympus CX43 microscope (Olympus, Tokyo, Japan). As the tumor epithelium was found to have immunoreactivity when CD4 was evaluated, only the cells in lymphocyte morphology were counted.

### 2.4. Statistical analysis

Statistical analyses were carried out using SPSS for Windows, version 17 (SPSS, Chicago, IL, USA). Shapiro-Wilk test was employed to determine whether or not the variables demonstrate a normal distribution. Student t-test was used for comparison of normally distributed data. The Mann-Whitney U test was utilized to analyze the data with abnormal distribution. Pre and postoperative comparisons (in-group) were performed suing the Wilcoxon rank sum test. Categorical variables were compared using the Chi-square test. P < 0.05 was regarded as significant.

## 3. Results

### 3.1. Clinical findings

Longitudinal data was available for 30 locally advanced rectal cancer (LARC) patients who had nCRT followed by surgical resection. Thirty patients were included in our study. Fifteen of these patients were in the group that received immunonutrition, and 15 of them were in the group that received standard nutrition. In the preoperative evaluation of both groups, the NRS 2002 score of 12 patients in the immunonutrition group was found to be below 3, and 3 or higher in 3 patients. In the group receiving standard nutritional supplements, the NRS 2002 score was found to be less than 3 in 13 patients and equal to or above 3 in 2 patients. No difference was found between the two groups in the NRS 2002 score evaluation (p = 0.624). When the preoperative BMI of the patients was evaluated, it was found to be 27.2 (20.01–30.06) (Mean 27.19 ± 1.04 SD) kg/m2 in the group receiving immunonutrition, and 24.6 (23.82–25.3) (Mean 24.8 ± 0.47 SD) kg/m2 in the group receiving standard nutritional supplement and there was no statistical difference between the two groups (p = 0.110). Open surgery was performed in 11 of the patients in the immunonutrition group and laparoscopic surgery was performed in 4 of them. Of the patients who received standard nutritional supplements, 12 underwent open surgery and 3 underwent laparoscopic surgery. There was no statistical difference between the two groups’ frequency of open and laparoscopic surgery (p = 0.666). The groups were similar with regard to age, gender distribution, type of surgery, distribution of differentiation, T stage, N stage, or TNM stages. Demographic information and histopathological characteristics of the patients are given in Table 1.

The immunonutrition and standard nutritional supplement groups were also similar concerning the number of lymph nodes removed and the number of metastatic lymph nodes (p = 0.569). The number of lymph nodes extracted was 14.7 (SD ± 6.95) in all the patients who received and did not receive immunotherapy (14.93, SD ± 8.54 and 14.46 SD ± 4.49, respectively).

### 3.2. Pathological findings and immunohistochemical results

The immunonutrition and standard nutrition groups were similar regarding CD4[10(5–20) vs. 10(10–10), p = 0.653, CD8[30 (20–35)] vs. 30 (20–40), p = 0.870], lymphocyte counts [2 (2–3) vs. 2 (2–3), p = 0.325], fox p3 value [10 (10–10) vs. 10 (10–10), p = 0.775], and CD4/CD8 ratio [0.33 (0.29–0.66) vs. 0.50 (0.29–0.50), p = 0.870] on endoscopic biopsy. Histopathological evaluations of preoperative colonoscopic and postoperative tumor specimens are given in Table 2. CD4 [10 (7.5–25) vs. 30 (10–50), p = 0.050], CD8 [60 (40–60) vs. 50 (40–60), p = 0.713] and Fox P3 [10 (5–10) vs. 10 (2.5–10), p = 0.935] were also similar in tissues extracted by surgery (Figures 2–3). However, the standard nutrition group had significantly higher CD4/CD8 values in their tissues removed on surgery [0.25 (0.14–0.50) vs. 0.66 (0.28–1), p = 0.026] (Table 2). Comparison of pre and postoperative intraepithelial lymphocyte count between the groups is presented in Table 3. Comparison of preoperative and postoperative nutritional condition is given in Table 4. The groups were similar with regard to serum prealbumin and albumin levels measured prior to and subsequent to surgery (Table 4).

Regarding postoperative complications, one patient developed a CD 1 complication and two patients developed CD 2 complications in the immunonutrition group according to Clavien-Dindo classification (CD). One of these patients developed atelectasis, one patient had wound infection, one patient had urinary retention (followed by urinary catheter for three weeks), and one patient had postoperative ileus on the 7^th^ postoperative day and resolved with follow-up. In the standard nutrition group, one patient developed a CD 1 complication, and four patients developed CD 2 complications. Of these patients, one had atelectasis, one urinary tract infection, one wound infection, one urinary retention, and one ileus (1 month later).

## 4. Discussion

The present study shows that immunonutiriton prior to surgery provides similar CD4 and CD8 T cell counts and FOXp3 levels compared to standard nutrition in locally advanced rectal cancer patients. However, CD4/CD8 ratios were lower in the immunonutrition group than the group receiving standard nutrition before surgery.

Cancer-related inflammation, emerging in any phases of tumorigenesis, plays a role in genetic alterations, altered gene expression, initiation of cancer cell growth, enhancement of cancer anti-apoptotic pathways, provoking angiogenesis, and ultimately cancer spread [18]. Research performed in the past 20 years have shown that inflammatory immune cells have crucial roles in inflammation linked with cancer. Therefore, attempts have concentrated on explaining the mechanism through which immune cells affect tumor outlook in different phases of cancer: initial neoplastic changes, clinically diagnosed tumors, metastatic spread, and therapeutic attempts [19]. CD8+ T cells are the most prominent antitumor cells and are prepared and activated by antigen-presenting cells. CD8+ T cells differentiate into cytotoxic T lymphocytes (CTLs) and, through exocytosis of perforin and granzyme-containing granules, carry out an effective antitumor attack resulting in direct destruction of target cells [20].

The intensity of tumor-infiltrating T cells, especially CD8 T cells is strongly suggestive of surviving without morbidity in colorectal cancer and rectal cancer [21]. CD8 T cell intensity on cancer biopsies is predictive of the probability for tumor remission after CRT [22]. A study conducted by Klintrup et al. on 372 patients operated on for colorectal cancer showed that low-grade inflammatory infiltration at the tumor’s invasive margin was significantly correlated with poor survival [23]. Roxburgh et al. revealed connection between the degree of TILs and cancer-specific survival in patients undergoing resection to cure colorectal cancer [24]. Huh et al. [25], also showed that the degree of TILs has a parallel relationship with survival, as in the studies by Klintrupet al. [23], and Roxburgh et al. [24]. The study performed by Shinto et al. showed that disease-free survival was better in patients with high CD 8 Cytotoxic T lymphocytes in postoperative evaluations [26]. Advocating the opposite, Morris and colleagues showed that TILs in stage 2 colon cancers have no prognostic value in their study [27]. In a study conducted by Caglayan et al., all colorectal cancers were evaluated [28]. They showed an increase in TILs in all feeding groups by comparing the specimen and preoperative biopsies. The same study emphasized that CD 8 positivity increased more significantly in the group receiving immunonutrition [28]. In the present study, the CD 8 numbers were not significantly different between the immunonutrition and standard nutrition groups.

The ratio of CD4/CD8 T cells has been utilized as a predictor to evaluate the immune system’s role. Although several studies have demonstrated that CD4 and CD8 have an immunological anti-tumor effect, there have been studies reporting the clinical importance of the CD4/CD8 ratio in tumor-infiltrating lymphocytes and their prognosis as a marker of the gastrointestinal tumor showing progression [29,30]. Diederichsen et al. showed an inverse relationship between the low CD4/CD8 ratio in tumor-infiltrating lymphocytes as an independent predictor of prognosis and survival in cases of colorectal carcinoma [29]. In their study, CD4+ TILs were predominant at the mean CD4/CD8 ratio of 2.2, and cases having low CD4/CD8 ratios were shown to have a more favorable prognosis [29]. Waki et al. found in their study on personalized peptide vaccination for ovarian cancers that the CD4/CD8 ratio was significantly related to the CTL reaction and CD8 T cells [31]. In fact, half of the cases with a lower CD4/CD8 ratio had a CTL reaction to vaccination peptides, while the cases with a higher CD4/CD8 ratio did not display a CTL reaction. Likewise, 66.7% of the cases with a lower CD4/CD8 ratio had higher levels of CD8 T cells; however, only 9.1% of the cases with a higher CD4/CD8 ratio had increased levels of CD8 T cells. In a study conducted by Peker et al. on the effect of immunonutrition on TILs in gastric cancers, there was no significant difference between CD 4 and CD 8 rates in both the patients given immunonutrition and those not given immunonutrition, and the CD4/CD 8 ratio was found to be significantly lower in the immunonutrition group [14]. In Flux et al.’s study, decreased CD4^+^/CD8^+^ and Foxp3^+^/CD8^+^ ratios correlated with a more favorable outcome in cases of gallbladder cancer, especially those with advanced disease [30]. Their finding shows that the existence of CD4^+^ T cells is not adequate to propose a tumor in a milieu containing decreased CD8^+^ T cells and that Foxp3+ T cells might have a negative effect on the potential of CD8^+^ T cells for fighting against gallbladder cancer. In the current study, the CD4/CD8 ratio was lower in the cases receiving immunonutrition (p = 0.026). Taking the evidence derived from the patients with gall bladder cancer into account, we speculate that immunonutrition may be associated with a more favorable outcome compared to standard nutrition owing to its impact on CD4^+^/CD8^+^ ratio.

Treg cells refer to CD4+ T lymphocytes playing an essential part in the immunological self-tolerance and immune homeostasis in the course of both abnormal and benign immunological reactions [32]. They release some part of the transcription factor Foxp3, needed in their development and suppressive roles [32]. Studies show a connection between the presence of a high number of FOXP3-positive T cells in tumor tissue and a higher potential of disease relapses and overall poor quality of life [33]. On the other hand, studies report that higher rates of FOXP3+ infiltrating tumor are associated with better patient survival [12,34]. Chew et al. found that higher rates of FOXP3-positive T cells significantly and strongly correlated with a good disease outcome, especially in stage II CRC [35]. In the same study, FOXP3 expression was higher in stage III CRC in patients with a good prognosis than in those with a poor prognosis, but it was not statistically significant [35]. In our study, when the immunonutrition and standard nutrition groups were compared, no statistical difference was found between their FOXP3 values (p = 0.935).

The limitations of our study are that nutritional follow-up could not be performed immediately after the diagnosis, and the presence of sarcopenia could not be evaluated except for anthropometric measurements. Another limitation of our study is the inability to monitor the amount of oral nutrition and oral nutritional supplement intake and to observe the changes in anthropometric measurements by following the patients just before they are diagnosed and sent to neoadjuvant chemoradiotherapy. The absence of survival data, small sample, and the lack of blood flow cytometry analysis are other limitations to be mentioned.

In conclusion, the present study revealed that CD4/CD8 ratios were lower in the immunonutrition group in comparison to the group receiving standard nutritional supplement before surgery. Although there is no significant change in the number of TILs, it can be proposed that immunonutrition provides a balance between TILs and Tregs in rectal cancer. However, studies including larger sample sizes and five-year survival are needed to confirm these findings.

## Figures and Tables

**Table 1:** Demographic and histopathological features of the study groups.

	Immunonutrition n = 15	Standard nutrition n = 15	P-Value

**Age**(years)	62.4 ±2.47	57.9 ±1.82	0.156*

**Gender**			
• Male	8 (53.3%)	9 (60%)	0.713**
• Female	7 (46.7%)	6 (40%)	

**Surgical procedure**			0.283**

Low-anterior resection. n	12 (80%)	14 (%93)	

Abdominopelvic resection. n	3 (20%)	1 (7%)	

**Differentiation**			0.224**

Well. n	2 (13.3%)	0	

Moderate. n	12 (80%)	15 (100%)	

Poor. n	1 (6.6%)	0	

**Lymphovasscular invasion**			0.099**

(+). n	6 (40%)	2 (13.3%)	

(−). n	9 (60%)	13 (86.7%)	

**Perineural invasion**			0.195**

(+). n	2 (13.3%)	5 (33.3%)	

(−). n	13 (86.6%)	10 (66.6%)	
	
**T classification**			0.417**

Total response. n	3 (20%)	1 (6.7%)	

T1. n	1 (6.7%)	1 (6.7%)	

T2. n	2 (13.3%)	4 (26.7%)	

T3. n	7 (46.7%)	9 (60%)	

T4. n	2 (13.3%)	0 (0)	
	
**N classification**			0.697**

N0. n	8 (53.3%)	10 (66.6)	

N1. n	2 (13.3%)	2 (13.3)	

N2. n	5 (33.3%)	3 (20)	

**Stage**			0.260**

1. n	6 (40%)	3 (20%)	

2A. n	3 (20%)	7 (46.7%)	

2B. n	0 (0%)	0 (0%)	

3A. n	0 (0%)	0 (0%)	

3B. n	6 (40%)	5 (33.3%)	

* P-value derived from student t-test

** P-value derived from chi-square test

**Table 2:** Histopathological evaluation of preoperative colonoscopic and postoperative tumor specimens.

	Immunonutrition n = 15	Standard nutrition n = 15	P-value^**^
CD4, preoperative	10 (5–20)	10 (10–10)	0.653
CD4, postoperative	10 (7.5–25)	30 (10–50)	0.050
P value^*^	0.916	**0.005**	
CD8, preoperative	30 (20–35)	30 (20–40)	0.870
CD8, postoperative	60 (40–60)	50 (40–60)	0.713
P value^*^	**0.002**	**0.003**	
CD4/CD8, preoperative	0.33 (0.29–0.66)	0.50 (0.29–0.50)	0.870
CD4/CD8, postoperative	0.25 (0.14–0.50)	0.66 (0.28–1)	**0.026**
P value^*^	0.158	0.231	
Foxp3, preoperative	10 (10–10)	10 (10–10)	0.775
Foxp3, postoperative	10 (5–10)	10 (2.5–10)	0.935
P-value^*^	**0.010**	**0.031**	
Lymphocyte count (tumor stouma), preoperative	2 (2–3)	2 (2–3)	0.325
Lymphocyte count (tumor stouma), postoperative	2 (2–2)	2 (2–3)	0.325
P-value^*^	0.366	0.206	

P value^*^: Wilcoxon rank sum test

P value^**^: Mann Whitney U test

**Table 3:** Comparison of pre and postoperative intraepithelial lymphocyte count between the groups.

	Immunonutrition n = 15	Standard nutrition n = 15	P-value*
Intraepithelial lymphocyte count, preoperative, n			
Moderate-common	8 (%53.3)	2 (%13.3)	0.316
Common-mild	2 (%13.3)	5 (%33.3)	
Focal-mild	5 (%33.3)	5 (%33.3)	
None	0 (%0)	3 (%20)	
Intraepithelial lymphocyte count, postoperative, n			
Moderate-common	2 (%13.3)	0	0.157
Common-mild	5 (%33.3)	7 (46.6%)	
Focal-mild	5 (%33.3)	8 (%53.3)	
None	3 (%20)	0	

* P value derived from chi-square test

**Table 4:** Comparison of preoperative and postoperative nutritional condition.

	Immunonutrition n = 15	Standard nutrition n = 15	P value^*^
Preoperative 7th day Prealbumin(mg/dL)	23 (20–24)	23 (20–25)	0.691
Preoperative 1st day Prealbumin(mg/dL)	24 (22.5–26)	23 (22–25)	0.558
Postoperative 2nd day Prealbumin(mg/dL)	17 (12–19.5)	14 (12–14.5)	0.086
Postoperative 7th day Prealbumin (mg/dL)	19 (16–22.5)	23 (20–24)	0.100
Preoperative 7th day Albumin(g/L)	4.1 (3.8–4.35)	4,1 (3.95–4.3)	0.464
Postoperative 7th day Albumin(g/L)	3.5 (3.2–3.75)	3.6 (3–3.8)	0.358

P-value^*^: Mann Whitney U test
